# Medicaid Accountable Care Organization Implementation and Behavioral Health Care for Children

**DOI:** 10.1001/jamanetworkopen.2026.8890

**Published:** 2026-04-23

**Authors:** Chanup Jeung, Sarah L. Goff, Jessica Pearlman, Barry Sarvet, Kimberley H. Geissler

**Affiliations:** 1 Department of Health Policy, Management and Behavior, College of Integrated Health Sciences, State University of New York at Albany; 2 Department of Health Promotion and Policy, University of Massachusetts Amherst School of Public Health and Health Sciences; 3 Institute of Social Science Research, University of Massachusetts Amherst; 4 Department of Psychiatry, University of Massachusetts Chan Medical School—Baystate, Springfield; 5 Department of Healthcare Delivery and Population Sciences, University of Massachusetts Chan Medical School—Baystate, Springfield

## Abstract

**Importance:**

Medicaid covers almost 40% of US children; state Medicaid programs are increasingly implementing financing and delivery system reforms through Accountable Care Organizations (ACOs). Little is known about how Medicaid ACOs are associated with outcomes for children with behavioral health conditions.

**Objective:**

To examine the association between Medicaid ACO implementation and behavioral health care access, unmet mental health needs, and care experience for Medicaid-insured children with behavioral health conditions.

**Design, Setting, and Participants:**

This repeated cross-sectional study used data from the 2016–2023 National Survey of Children’s Health, a nationally representative household survey of US children. The study included children aged 3 to 17 years insured by Medicaid whose caregivers reported at least 1 current behavioral health condition (anxiety, depression, attention-deficit/hyperactivity disorder, Tourette syndrome, or behavioral problems), with analyses conducted between January 2025 and February 2026. Using a difference-in-differences design, changes in outcomes before and after Medicaid ACO implementation in states that implemented Medicaid ACOs between 2017 and 2022 were compared with contemporaneous changes in states without Medicaid ACO implementation.

**Exposure:**

Medicaid ACO implementation.

**Main Outcomes and Measures:**

Primary outcomes were (1) having a personal physician or nurse, (2) receiving treatment from a mental health professional in the past 12 months, (3) experiencing unmet mental health needs, (4) receiving effective care coordination, and (5) receiving family-centered care.

**Results:**

The sample represented 29 885 590 children (unweighted n = 15 783; females, 41.0%), and 9.1% resided in states with Medicaid ACO implementation. Medicaid ACO implementation was associated with higher reported unmet mental health needs (4.76 percentage points; 95% CI, 0.83–8.70 percentage points). Medicaid ACO implementation was not associated with having a personal physician or nurse, treatment from a mental health professional, care coordination, or family-centered care.

**Conclusions and Relevance:**

In this cross-sectional difference-in-differences study of Medicaid-insured children with behavioral health conditions, Medicaid ACO implementation was not associated with broad improvements in children’s behavioral health care and was associated with higher unmet needs. These findings suggest that pediatric-specific strategies and workforce investments are needed in Medicaid ACOs to support children with behavioral health conditions.

## Key Points

**Question:**

Is implementation of Medicaid Accountable Care Organizations (ACOs) associated with changes in behavioral health care access and quality for children with behavioral health conditions?

**Findings:**

In this cross-sectional study including 15 783 Medicaid-insured children (representing 29 885 590 nationally) with behavioral health conditions, Medicaid ACO implementation was not associated with improvements in behavioral health care access or care experience. ACO implementation was associated with an increase in unmet mental health needs.

**Meaning:**

Results of this study suggest that Medicaid ACOs have not yet produced broad gains in behavioral health care for children and may exacerbate unmet needs, underscoring the need for pediatric-specific strategies within ACO models.

## Teaser

This cross-sectional study reports on whether the implementation of Medicaid Accountable Care Organizations is associated with changes in behavioral health care access and quality for children with behavioral health conditions, unmet mental health needs, and care experience.

## Introduction

Improving access to care and health outcomes for persons with behavioral health (BH) conditions has become a central focus of US Medicaid delivery system reform, particularly as Medicaid is the largest payer for BH services in the US.^1,2,3^ This emphasis is particularly important for children as nearly 40% of children are covered by Medicaid.^4^ Many with BH needs face persistent barriers to timely, coordinated, and effective care.^5,6,7,8^ These challenges are often compounded by complex social needs for families, mental health professional shortages overall and for pediatrics specifically, and limited integration between medical and BH care.^9,10,11,12,13^ Traditional fee-for-service arrangements have historically struggled to address these challenges, reinforcing reactive and episodic care.^14^

To address these issues, many states have adopted Medicaid Accountable Care Organizations (ACOs) to realign financial incentives toward value and quality. Medicaid ACOs increase incentives to coordinate care, improve quality, and contain costs through shared savings, performance measurement, and care management infrastructure.^15,16,17,18,19,20,21,22^ States often integrate BH within their ACO models^23,24^ with approaches such as enhanced payment incentives for BH services,^23,25^ BH-specific quality metrics tied to financial incentives,^23,26^ and rewards for embedding BH professionals into care teams.^23,25^ Some states have also invested in workforce development,^25,27,28^ data-sharing infrastructure,^25,28^ and cross-sector partnerships to support cohesive care delivery,^23,29^ all of which may improve BH care. For children with BH needs, Medicaid ACOs may better integrate BH services within routine pediatric care, strengthen care continuity, reduce reliance on costly emergency or inpatient care, and conduct additional screening and linkages for health-related social needs^30,31,32^; however, the way in which Medicaid ACO programs incentivize changes in care delivery varies substantially across states, including inclusion of BH metrics and inclusion of pediatric members. Thus, it is unclear whether Medicaid ACOs—often focused on costlier adult populations—benefit children or require additional efforts to specifically address pediatric BH care.^33^

Despite growing policy interest, evidence on how Medicaid ACOs change BH care delivery for children remains limited. Most existing research has centered on general pediatric outcomes rather than BH-specific measures.^31,32,34^ One recent national study found modest gains in having a personal physician or nurse following ACO implementation without consistent improvements across other pediatric domains.^31^ Another study of children with disabilities reported mixed outcomes, including decreases in home health use, an important support for populations with high needs.^35^ Early evaluation of Oregon Medicaid ACOs found improvements in pediatric preventive care,^17^ and a study of Medicaid ACO programs from Massachusetts, Maine, Minnesota, and Vermont reported gains in pediatric developmental screenings.^18^ These findings highlight the need for further evidence on Medicaid ACO effectiveness for children’s BH.

Using nationally representative data, we examined whether Medicaid ACO implementation was associated with changes in BH care access and quality for Medicaid-insured children with BH conditions. We hypothesized that, by incentivizing care coordination, integration, and value-based delivery, Medicaid ACOs would improve access to care and care experiences for Medicaid-insured children with BH needs.

## Methods

### Data and Sample

This repeated cross-sectional study used data from the National Survey of Children’s Health (NSCH), an annual nationally representative survey on the health and well-being of US children. We used NSCH data from 2016 (first year of comparable data) through 2023, covering periods before and after Medicaid ACO implementation in multiple states. The NSCH is administered by the US Census Bureau on behalf of the Health Resources and Services Administration Maternal and Child Health Bureau. The Data Resource Center for Child and Adolescent Health constructs composite variables we used as outcomes.^36^ Weighted response rates varied from 35.8% to 43.1%. The survey was offered in English and Spanish, and representation from English primary household languages ranged from 82.5% to 85.3% (90.8% [weighted] to 93.0% [unweighted]). Analyses were conducted between January 2025 and February 2026. This study was approved by the University of Massachusetts Amherst Institutional Review Board, with a waiver of informed consent, as the study used deidentified publicly available secondary data, and reported using the Strengthening the Reporting of Observational Studies in Epidemiology (STROBE) reporting guideline. The sample included children ages 3 to 17 years publicly insured through Medicaid or the Children’s Health Insurance Program (CHIP) whose caregiver reported the child currently had 1 or more of the following conditions: anxiety, depression, attention-deficit/hyperactivity disorder (ADHD), Tourette syndrome, or behavioral problem (including oppositional defiant disorder and conduct disorder). The sample restriction criteria are presented in the eFigure of Supplement 1.

### Exposure

The sample includes children residing in states that either implemented Medicaid ACOs during the study period or had not done so by 2022 (eMethods and eTable 1 in Supplement 1). Exposure was defined at the state-year level. Children residing in states that implemented Medicaid ACOs during the study period were considered exposed beginning in the implementation year and thereafter. Children in ACO states prior to implementation and children in all other nonimplementing states throughout the study period were considered unexposed.

### Outcome Measures

We focused on 5 key outcome measures: (1) having a personal physician or nurse; (2) receiving treatment from a mental health professional in the past 12 months; (3) experiencing unmet mental health needs, defined as caregiver report that the child needed mental health care or counseling in the past 12 months but did not receive it; (4) receiving effective care coordination (composite); and (5) receiving family-centered care (composite).^36^ Care coordination is based on whether a child received needed help arranging or coordinating care, whether the child’s physicians communicated with each other, and whether clinicians communicated effectively with schools or other programs. Family-centered care is defined by whether clinicians consistently spent enough time, listened carefully, respected family values, provided needed information, and treated parents as partners in care. These variables are constructed only when all required component items are answered (including ≥1 visit).

### Other Measures

Additional covariates included indicators for specific behavioral conditions (ADHD, behavioral problem, anxiety, depression, and Tourette syndrome) as well as reported condition severity (severe vs moderate/mild). Autism spectrum disorder was included as a covariate but was not used to define the analytic sample, as autism is typically managed through different service systems and insurance coverage mechanisms.^37^ We accounted for child demographic characteristics (age category, sex, race and ethnicity) and parental educational level. Child race and ethnicity was caregiver-reported using predefined NSCH categories and was included to account for potential disparities in behavioral health care access and outcomes.

### Statistical Analysis

Descriptive statistics were calculated and differences by Medicaid ACO status calculated using χ^2^ tests. To estimate associations between Medicaid ACO implementation and BH outcomes among Medicaid-insured children, we used a staggered difference-in-differences (DID) design using a 2-way fixed-effects linear probability model. This approach leverages variation in the timing of Medicaid ACO implementation across states to compare differences in outcomes before and after implementation between exposed and unexposed states. We first estimated an unadjusted model with state fixed effects to control for time-invariant state-specific heterogeneity and year fixed effects to control for secular trends. We then estimated an adjusted model additionally accounting for the covariates (eMethods in Supplement 1). Reported estimates represent percentage point (pp) differences associated with exposure to Medicaid ACO implementation.

A key underlying identification assumption is that outcomes in states with and without Medicaid ACOs followed common trends prior to ACO implementation. To assess this, we estimated event study models with event-time indicators interacted with the Medicaid ACO implementation indicator, using the year prior to implementation as the reference (eMethods in Supplement 1). As an additional identification check, we conducted falsification tests examining whether Medicaid ACO implementation was associated with having any BH condition among Medicaid-enrolled or CHIP-enrolled children or with condition severity. To assess whether Medicaid ACO implementation was associated with differential outcomes by clinical complexity, we next estimated subgroup-specific DID associations by interacting the DID term with an indicator for single vs multiple BH conditions and used Wald tests to assess differences between subgroups.

We conducted several sensitivity analyses. First, methodologic studies have highlighted potential bias in conventional staggered DID models, particularly when treatment timing varies across units and changes evolve over time.^38^ In this study, states implemented Medicaid ACOs in different years, creating a setting where later-implementing states might inadvertently serve as controls for earlier-implementing states and distort estimated results.^38^ To address this concern, we used a 2-stage DID estimator^39^ to assess the robustness of our primary staggered DID results. In the first stage, we residualized each outcome by regressing it on the full set of covariates and on state and year fixed effects using only observations from untreated states. The residuals from this regression represent the portion of outcome variation not explained by observed characteristics or common time trends and were used as the dependent variable in the second stage. In that stage, we regressed the residuals on an indicator for the postimplementation period. The resulting coefficient captures differences in outcomes in the postimplementation period relative to preimplementation outcomes and contemporaneous outcomes in unexposed states.

Second, we excluded condition severity, which could differ based on changes in care received. Third, we included state-specific linear time trends to account for potential differences in the magnitude or patterns of preperiod estimates across states. Fourth, we excluded the first implementation year for each state to account for potential differences in survey vs implementation timing. Fifth, we excluded observations from 2020 and 2021 to account for COVID-19–related impacts. Sixth, we used alternative variance estimators (state-clustered SEs and wild cluster bootstrap). Finally, to address Medicaid ACO design heterogeneity, we excluded Idaho, where BH services are not included in total cost of care accountability, and New Jersey, whose model does not include formal shared savings or downside risk. We also estimated models excluding both states.

Analyses accounted for complex survey design and nonresponse. Statistical significance was defined as a 2-sided *P* < .05. Analyses were conducted using Stata/MP version 18 (StataCorp LLC).

## Results

Our analytic sample included 15 783 children (weighted n = 29 885 590; females, 41.0%) with BH conditions (Table 1). Of these, 9.1% resided in states that implemented Medicaid ACOs during the period and 90.9% in states that did not. Children in states with and without Medicaid ACOs were broadly similar across most demographic characteristics and prevalence of autism spectrum disorder. However, children in states with Medicaid ACOs were more likely to have parents with a college degree or higher (30.6% vs 22.2%) and were less likely to be non-Hispanic Black (12.9% vs 22.4%).

**Table 1.** Sample Characteristics^a^

Characteristic	Overall, %	States without Medicaid ACOs, %	States with Medicaid ACOs, %	*P* value
Sample, unweighted No.	15 783	12 994	2789	NA
Population, weighted No.	29 885 590	27 164 327	2 721 263	NA
Outcomes				
Had a personal physician/nurse	73.4	73.0	77.3	.02
Received treatment from a mental health professional	46.3	45.9	50.7	.02
Unmet mental health needs	6.1	5.9	7.6	.12
Received coordinated care^b^	53.4	54.1	47.2	.003
Received family-centered care^b^	76.5	76.1	80.4	.01
Current behavioral health conditions				
Tourette syndrome	1.0	1.0	1.4	.31
Anxiety	46.7	46.2	50.9	.02
Depression	25.1	24.8	28.7	.03
Behavioral problem	53.9	54.0	53.1	.68
ADHD	57.8	58.2	54.4	.06
Child age category				
3-5 y	8.9	9.0	7.6	.46
6-11 y	42.5	42.5	42.9	
12-17 y	48.6	48.5	49.5	
Child sex				
Female	41.0	41.0	40.8	.94
Male	59.0	59.0	59.2	
Autism	17.9	17.6	20.0	.13
Parent educational level				
Less than high school	13.9	14.3	9.6	<.001
High school	32.2	32.4	30.6	
Some college	31.0	31.2	29.3	
College or higher	23.0	22.2	30.6	
Race and ethnicity^c^				
Hispanic	27.3	26.9	31.9	<.001
Non-Hispanic Black	21.5	22.4	12.9	
Non-Hispanic White	43.6	43.4	46.4	
Non-Hispanic other^d^	7.5	7.4	8.8	

Abbreviations: ACO, Accountable Care Organization; ADHD, attention-deficit/hyperactivity disorder; CHIP, Children’s Health Insurance Program; NA, not applicable.

^a^ Data are from the 2016-2023 National Survey of Children’s Health. All years are included, including years in which the ACOs had not yet been implemented. The sample is restricted to children aged 3-17 years insured by Medicaid or CHIP with at least 1 behavioral health condition (Tourette syndrome, anxiety, depression, behavioral problem, or ADHD). All estimates use population weights to make the sample nationally representative. *P* values were calculated using χ^2^ tests for binary and categorical variables.

^b^ Received coordinated care and family-centered care are composite measures derived from multiple survey items. Children were coded as missing for these outcomes if any component item was missing or skipped because of survey nonresponse or skip patterns, resulting in smaller analytic samples for these measures. For received coordinated care, the unweighted analytic sample sizes were 11 552 overall (weighted n = 20 934 774), 9426 in control states (weighted n = 18 985 186), and 2126 in treated states (weighted n = 1 949 587). For received family-centered care, the corresponding unweighted analytic sample sizes were 15 186 overall (weighted n = 29 016 088), 12 486 in control states (weighted n = 26 402 015), and 2700 in treated states (weighted = 2 614 073).

^c^ Child race and ethnicity was caregiver-reported using predefined National Survey of Children’s Health categories.

^d^ Non-Hispanic other includes children identified as Asian, American Indian or Alaska Native, Native Hawaiian or Other Pacific Islander, or 2 or more races.

With regard to BH conditions, children in states with Medicaid ACOs exhibited higher rates of anxiety (50.9% vs 46.2%; *P* = .02) and depression (28.7% vs 24.8%; *P* = .03) but lower rates of ADHD (54.4% vs 58.2%; *P* = .06) with no differences in behavioral problems. Children in states with Medicaid ACOs were more likely to have a personal physician or nurse (77.3% vs 73.0%; *P* = .02), to have received treatment from a mental health professional (50.7% vs 45.9%; *P* = .02), and to receive family-centered care (80.4% vs 76.1%; *P* = .01). Conversely, they were less likely to receive coordinated care (47.2% vs 54.1%; *P* = .003). Because Medicaid ACO implementation was staggered, simple pre-post comparisons do not account for adoption timing differences. To assess baseline comparability and contextualize DID estimates, we report preimplementation outcome rates in states with and without Medicaid ACOs (eTable 2 in Supplement 1).

Event studies indicated that preimplementation coefficients were centered around 0 and did not exhibit preperiod systematic upward or downward trends for all outcomes (Figure). While individual preperiod estimates vary in magnitude, particularly for measures with wider CIs, we did not observe consistent directional patterns suggestive of differential pretrends. In falsification tests, we did not observe changes in the probability of having a BH diagnosis or in condition severity associated with Medicaid ACO implementation (eTable 3 in Supplement 1).

**Figure.**
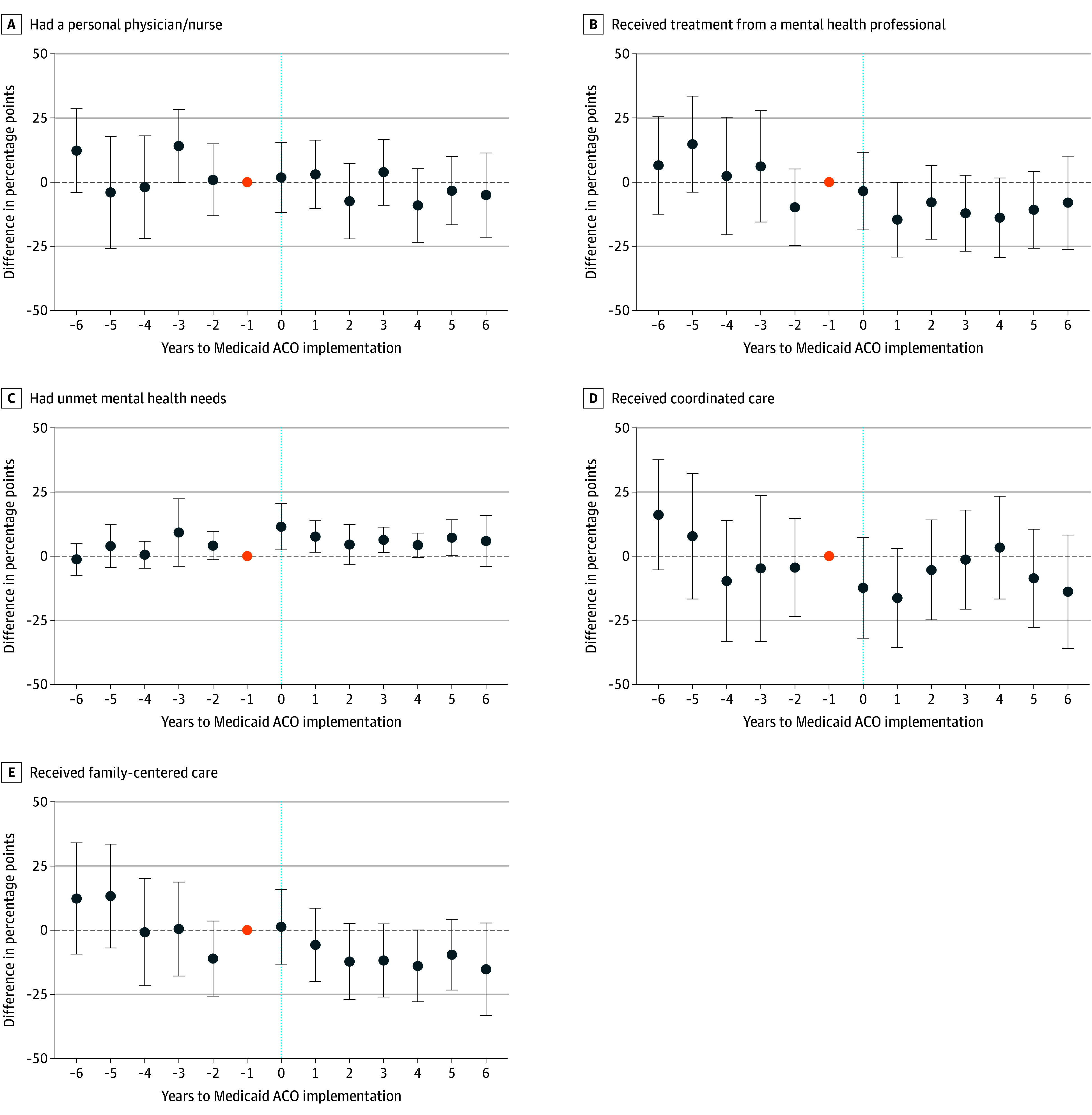
Dot Plots Showing Event Study Estimates for Behavioral Health Access, Unmet Need, and Care Experience Among Medicaid-Insured Children With Behavioral Health Conditions Each point represents the annual difference in outcomes between states that adopted Medicaid ACOs and those that did not, relative to 1 year before Medicaid ACO implementation (event year = -1), which serves as the reference period. The orange dot indicates the omitted reference category (event year = -1) with an estimate of 0. Estimates are from a fully adjusted survey-weighted model. Error bars indicate 95% CIs. The vertical dotted line marks the year of Medicaid ACO implementation (event year = 0). ACO indicates Accountable Care Organization.

In regression-adjusted staggered DID estimates, Medicaid ACO implementation was associated with higher reported unmet mental health needs (4.76 pp; 95% CI, 0.83-8.70 pp) (Table 2). Unadjusted results were similar (eTable 4 in Supplement 1). No associations were found for having a personal physician or nurse, receiving treatment from a mental health professional, coordinated care, or family-centered care (Table 2). For interpretability, eTable 5 in Supplement 1 reports preimplementation baseline outcome rates and corresponding relative changes for exposed states.

**Table 2.** DID Estimates of Medicaid ACO Implementation Association With Behavioral Health Access, Unmet Need, and Care Experience Outcomes^a^

DID estimate type	Outcome, percentage-point difference (95% CI)				
	Had a personal physician/nurse	Received treatment from a mental health professional	Unmet mental health needs	Received coordinated care	Received family-centered care
Overall^b^					
ACO × post	−2.48 (−10.10 to 5.14)	−7.27 (−15.53 to 0.98)	4.76 (0.83 to 8.70)^c^	−5.17 (−15.57 to 5.22)	−0.46 (−8.29 to 7.37)
Differences by condition complexity^d^					
ACO × post × single condition	−1.29 (−10.34 to 7.76)	−9.45 (−18.58 to −0.32)^c^	3.25 (−0.32 to 6.82)	−3.98 (−15.95 to 7.99)	0.70 (−7.99 to 9.39)
ACO × post × multiple conditions	−3.42 (−11.66 to 4.81)	−5.55 (−14.55 to 3.44)	5.96 (0.57 to 11.35)^c^	−5.86 (−16.91 to 5.18)	−1.30 (−9.87 to 7.27)
Wald test *P* value for differences between coefficients	0.602	0.310	0.299	0.704	0.869
No. of observations	15 783	15 783	15 783	11 552	15 186
Weighted population, No.	29 885 590	29 885 590	29 885 590	20 934 774	29 016 088

Abbreviations: ACO, Accountable Care Organization; ADHD, attention-deficit/hyperactivity disorder; DID, difference-in-differences; NSCH, National Survey of Children’s Health; post, postimplementation.

^a^ Data from the 2016-2023 NSCH. Linear probability models were estimated, controlling for child age (categorical), sex, race and ethnicity, autism, specific behavioral health condition (Tourette syndrome, anxiety, depression, behavioral problem, ADHD), and severity for each (severe vs not severe), as well as year and state fixed effects. Reported estimates are expressed in percentage points, and all models are weighted to ensure national representativeness. Sample sizes differ across outcomes because coordinated care and family-centered care are composite measures constructed only for children who met NSCH eligibility criteria (eg, having at least 1 health care visit and complete responses to all required items). Positive coefficients indicate higher probability of the outcomes (in percentage points) in states with Medicaid ACOs relative to states without Medicaid ACOs, whereas negative coefficients indicate a lower probability.

^b^ Overall DID estimates of Medicaid ACO implementation.

^c^ *P* < .05.

^d^ Subgroup-specific DID estimates by behavioral health condition complexity, obtained by interacting the DID indicator with indicators for having a single vs multiple conditions. These coefficients represent subgroup-specific DID associations compared with contemporaneous changes in states without Medicaid ACO implementation.

We next decomposed overall DID estimates by BH condition complexity by interacting the DID term with an indicator of having a single vs multiple conditions (Table 2). For unmet mental health needs, Medicaid ACO implementation was not associated with a significant difference among children with a single condition (3.25 pp; 95% CI, −0.32 to 6.82 pp) but was associated with a significant difference among children with multiple conditions (5.96 pp; 95% CI, 0.57-11.35 pp). For receipt of treatment from a mental health professional, implementation was associated with a significant difference among children with a single condition (−9.45 pp; 95% CI, −18.58 to −0.32 pp) but was not associated with significant difference among children with multiple conditions (−5.55 pp; 95% CI, −14.55 to 3.44 pp). Wald tests showed that subgroup coefficients for both outcomes did not differ significantly, implying no statistical evidence of heterogeneity despite differences in significance levels. No other outcomes showed associations in either subgroup.

In the first sensitivity analysis using the 2-stage DID model, results were consistent with the main DID estimates (Table 3). Medicaid ACO implementation was associated with higher reported unmet mental health needs (4.84 pp; 95% CI, 1.11-8.57 pp). No associations were found for other outcomes in either model. Results were generally consistent across sensitivity analyses (eTables 4 and 6 in Supplement 1). Excluding the first implementation year to account for the 12-month look-back period in NSCH outcomes and survey years 2020-2021 yielded larger reductions in treatment from a mental health professional. Excluding the first implementation year, the estimates were not statistically significant. Results were also similar when excluding Idaho, New Jersey, or both states, which represent structurally distinct Medicaid ACO models (eTable 7 in Supplement 1). The estimates for unmet mental health needs remained positive and statistically significant across these specifications.

**Table 3.** Two-Stage DID Estimates of Medicaid ACO Implementation Association With Behavioral Health Measures^a^

Estimate type	Outcome, DID (95% CI)				
	Had a personal physician/nurse	Received treatment from a mental health professional	Unmet mental health needs	Received coordinated care	Received family-centered care
Medicaid ACO DID coefficient	−4.14 (−11.79 to 3.50)	−6.93 (−17.12 to 3.27)	4.84 (1.11 to 8.57)^b^	−3.74 (−10.58 to 3.09)	−3.19 (−9.61 to 3.23)
No. of observations	15 783	15 783	15 783	11 552	15 186

Abbreviations: ACO, Accountable Care Organization; ADHD, attention-deficit/hyperactivity disorder; DID, difference-in-differences.

^a^ Data were obtained from the 2016-2023 National Survey of Children’s Health. Linear probability models were estimated, controlling for child age (categorical), sex, race and ethnicity, autism, specific behavioral health condition (Tourette syndrome, anxiety, depression, behavioral problem, ADHD), and severity for each (severe vs not severe), as well as year and state fixed effects. Reported estimates are expressed in percentage points, and all models are weighted to ensure national representativeness.

^b^ *P* < .05.

## Discussion

This cross-sectional study examined associations between Medicaid ACO implementation and BH care for children. Contrary to expectations, we did not find differential changes in access to a personal physician or nurse, receipt of treatment from a mental health professional, coordinated care, or family-centered care in states with Medicaid ACOs following implementation compared with changes in states without Medicaid ACOs. However, implementation was associated with higher reported unmet mental health needs.

These findings are consistent with broader national evidence showing that Medicaid ACOs have produced mixed or modest improvements in pediatric access and services use.^31,35^ Recent state-level Medicaid ACO evaluations reinforce this conclusion. One evaluation found no meaningful improvements in asthma care for children compared with privately insured children during the first 3 years of implementation.^40^ This may reflect perceived limited returns on investment for pediatric populations compared with adults, and less collaboration of pediatric practices within mixed-age Medicaid ACOs.^41^ Together, these studies highlight potential issues for children in the early years of Medicaid ACOs and underscore the need for stronger pediatric-specific strategies.

Multiple factors likely contribute to these modest or mixed results. Persistent workforce shortages, especially among pediatric BH clinicians, may restrict timely access despite Medicaid ACOs’ incentives to integrate BH care.^42^ Even well-structured ACOs and incentives to improve BH care may have difficulty in meeting the needs of children if clinician availability and referral networks are limited. Such barriers may help explain why we observed higher reported levels of unmet mental health needs in our analysis, particularly among children with multiple BH conditions.

Beyond statewide Medicaid ACO implementation, a 2023 evaluation of pediatric care management programs for children at high risk (such as those that might be used in an ACO), defined by medical complexity, multiple chronic conditions, or high prior utilization, but not BH specific, found reductions in inpatient and ED utilization but no consistent improvements in care coordination, family experience, or satisfaction.^32^ Even with this care management program, many families reported difficulty obtaining needed BH services and perceived ongoing unmet needs.^32^

Structural and organizational features of Medicaid ACOs may also help explain our findings. Unlike Medicare or commercial ACOs, where clinician participation is typically voluntary and based on market incentives, Medicaid ACO participation is often mandated or includes clinicians with less choice in engagement.^43,44,45^ This can result in the inclusion of clinicians who are less prepared or resourced to effectively participate, potentially reducing overall ACO efficiency. In addition, many state-led Medicaid ACO models, particularly those designed to serve safety-net populations, enter implementation with limited infrastructure, such as health information technology, data analytics, and care management capacity.^46,47^ Developing these capabilities is often a first step in ACO implementation regardless of payer types, but gaps at baseline may slow progress and hinder early performance. These structural and resource constraints may help explain why we did not observe favorable outcome patterns following Medicaid ACO implementation and suggest the need for longer-term evaluation.

### Limitations

This study has limitations. First, our analysis relied on caregiver-reported data, which may be subject to recall or reporting bias and may differ from clinical diagnoses. However, the NSCH is widely used in pediatric and health services research and its caregiver report measures of BH conditions are a core component of national child mental health surveillance.^48,49^ Second, we were limited to states that implemented Medicaid ACOs after 2016; thus, findings may not be generalizable to earlier-adopting states, which may differ in baseline infrastructure, policy capacity, or implementation maturity. Third, the NSCH lacks information on state-specific Medicaid ACO program characteristics, which may vary substantially. We identified key structural features of Medicaid ACO models using external policy sources. Although program design varies, results were robust to sensitivity analyses excluding states with structurally distinct Medicaid ACO models. Future research should examine differences in Medicaid ACO program impacts based on variation in program design, state-specific policies, resources and incentives, and workforce availability. Fourth, our outcomes are measured at the child level and do not capture system-level performance metrics such as clinician engagement or care management intensity, which could further explain the mechanisms underlying our findings. Fifth, our results have somewhat wide CIs for some outcomes, suggesting that we may be underpowered to detect small differences in care utilization patterns. Although the NSCH is designed to be population-representative at the national and state level, it may not be fully representative of Medicaid populations in states with smaller numbers of respondents. Finally, this is an observational study rather than a randomized evaluation. However, our DID design with state and year fixed effects helps limit bias from unobserved state characteristics and secular trends, including changes during the pandemic.

## Conclusions

In this repeated cross-sectional study of Medicaid-insured children with BH conditions, Medicaid ACO adoption, while conceptually promising, was not associated with broad improvements in BH care access or quality and was associated with higher reported unmet mental health needs. These findings, consistent with prior evidence showing mixed or null changes in outcomes with Medicaid ACO implementation, highlight the need for targeted strategies to strengthen BH integration, expand the pediatric BH workforce, and improve care coordination within Medicaid ACOs. Without such focused efforts, Medicaid ACOs may fall short of their potential to reduce unmet BH needs and improve outcomes for children with high needs.

## Notes

Supplement 1. **eMethods.** Exposure, Staggered Difference-in-Differences, and Event Study **eFigure.** Sample Restriction Criteria **eTable 1.** Medicaid ACO Program Characteristics **eTable 2.** Baseline (Pre-Medicaid ACO Implementation) Outcome Rates in States with and without Medicaid ACO **eTable 3.** Association between Medicaid ACO Implementation and Behavioral Health Diagnosis and Condition Severity **eTable 4.** Sensitivity Analyses **eTable 5.** Pre-Implementation Baseline Outcome Rates and Relative Percentage Changes Associated with Medicaid ACO **eTable 6.** Sensitivity Analysis: Difference-in-Differences Estimates using State-Clustered Standard Errors and Wild Cluster Bootstrap **eTable 7.** Sensitivity Analyses: Excluding Structurally Distinct Medicaid ACO Models (Idaho and/or New Jersey)

Supplement 2.
Data Sharing Statement
