# Supplementary material for: Medicaid Accountable Care Organization Implementation and Behavioral Health Care for Children
Source: JAMA Netw Open. 2026 Apr 23;9(4):e268890. doi: 10.1001/jamanetworkopen.2026.8890 (PMC13107223; doi:10.1001/jamanetworkopen.2026.8890)
Supplement: Supplement 1. — eMethods. Exposure, Staggered Difference-in-Differences, and Event Study eFigure. Sample Restriction Criteria eTable 1. Medicaid ACO Program Characteristics eTable 2. Baseline (Pre-Medicaid ACO Implementation) Outcome Rates in States with and without Medicaid ACO eTable 3. Association between Medicaid ACO Implementation and Behavioral Health Diagnosis and Condition Severity eTable 4. Sensitivity Analyses eTable 5. Pre-Implementation Baseline Outcome Rates and Relative Percentage Changes Associated with Medicaid ACO eTable 6. Sensitivity Analysis: Difference-in-Differences Estimates using State-Clustered Standard Errors and Wild Cluster Bootstrap eTable 7. Sensitivity Analyses: Excluding Structurally Distinct Medicaid ACO Models (Idaho and/or New Jersey) [file jamanetwopen-e268890-s001.pdf]

Supplemental Online Content

Jeung C, Goff SL, Pearlman J, Sarvet B, Geissler KH. Medicaid Accountable Care Organization implementation and behavioral health care for children. *JAMA Netw Open*. 2026;9(4):e268890. doi:10.1001/jamanetworkopen.2026.8890

eMethods. Exposure, Staggered Difference-in-Differences, and Event Study
eFigure 1. Sample Restriction Criteria
eTable 1. Medicaid ACO Program Characteristics
eTable 2. Baseline (Pre-Medicaid ACO Implementation) Outcome Rates in States with and without Medicaid ACO
eTable 3. Association between Medicaid ACO Implementation and Behavioral Health Diagnosis and Condition Severity
eTable 4. Sensitivity Analyses
eTable 5. Pre-Implementation Baseline Outcome Rates and Relative Percentage Changes Associated with Medicaid ACO
eTable 6. Sensitivity Analysis: Difference-in-Differences Estimates using State-Clustered Standard Errors and Wild Cluster Bootstrap
eTable 7. Sensitivity Analyses: Excluding Structurally Distinct Medicaid ACO Models (Idaho and/or New Jersey)

This supplemental material has been provided by the authors to give readers additional information about their work.

© 2026 Jeung C et al. *JAMA Network Open*.

eMethods

1. Exposure

The sample includes children residing in states that either implemented Medicaid ACOs during the study period or had not done so by 2022. States that implemented Medicaid ACOs in or before 2016 (Oregon, Ohio, Utah, New York, Maine, Iowa, and Connecticut) were excluded from the analysis. This exclusion was necessary because the 2016 redesign of the NSCH data serves as our earliest available data point, and these early-adopting states lack a sufficient pre-implementation baseline for our staggered DID design. Specifically, for states like Iowa which implemented in 2016, the data from that year would already reflect initial changes as a result of the policy, making it impossible to establish a clean pre-implementation period. Exposure was defined at the state-year level. Children residing in states that implemented Medicaid ACOs during the study period were considered exposed beginning in the implementation year and thereafter. The ACO implementation group includes Vermont (implemented in 2017), Colorado, Massachusetts, Minnesota, and Rhode Island (2018); New Jersey (2020); Delaware (2021); and Idaho (2022). Children in these states prior to implementation, and children in all other non-implementing states throughout the study period, were considered unexposed.

2. Staggered Difference-in-Differences

(1) Main Specification

Our primary empirical approach is a staggered difference-in-differences (DID) design that compares states with and without Medicaid ACO implementation. Because implementation

© 2026 Jeung C et al. *JAMA Network Open*.

occurred in different states at different times, we estimated the following linear probability model:

$$y_{ist} = \alpha + \beta \cdot ACO_s \cdot Post_{st} + X_{ist} \cdot \gamma + \delta_s + \eta_t + \varepsilon_{ist}$$

where y_{ist} denotes the outcome for child *i* in state *s* and year t; ACO_s is an indicator for states that adopted a Medicaid ACO; $Post_{st}$ is a post-implementation indicator for the treated state-year; X_{ist} is a vector of control variables as described above; δ_s are state fixed effects that control for time invariant state-specific heterogeneity; η_t are year fixed effects that control for contemporaneous shocks across states; and ε_{ist} is the error term. β captures the differential change in outcomes between states with and without Medicaid ACO implementation.

(2) Heterogenous Difference-in-Differences Specification

To examine heterogeneity in the effects of Medicaid ACO implementation by condition complexity (single vs. multiple behavioral health conditions), we extended the primary DID specification to include an interaction between the DID term (state × post-implementation) and mutually exclusive subgroup indicators defined by whether a child had a single or multiple behavioral health conditions. Specifically, we estimated the following DID model:

$$y_{ist} = \alpha + \beta_1(ACO_s \cdot Post_{st} \cdot Single_{ist}) + \beta_2(ACO_s \cdot Post_{st} \cdot Multiple_{ist})$$
$$+X_{ist} \cdot \gamma + \delta_s + \eta_t + \varepsilon_{ist}$$

where, $Single_{ist}$ and $Multiple_{ist}$ represent subgroup-specific DID effects, the first capturing the change in outcomes among children with single BH condition in ACO-implementing states relative to non-ACO states, and the second capturing this change in outcomes for those with multiple BH conditions. This approach uses this interaction term to estimate the overall DID

© 2026 Jeung C et al. *JAMA Network Open*.

effect by condition complexity. β_1 and β_2 capture the unique association of Medicaid ACO implementation with outcomes for each respective subgroup.

3. Event Study

We tested the common trend assumption for outcome measures between states with and without Medicaid ACO implementation. By interacting a dummy of Medicaid ACO with relative year dummies (i.e., relative year to the reference year: one year prior to the Medicaid ACO implementation), the following specification was estimated:

$$y_{ist} = \alpha + \sum_{t=-6}^{-2} \beta_t \cdot ACO_s \cdot Year_t + \sum_{t=0}^{6} \beta_t \cdot ACO_s \cdot Year_t + X_{ict}\gamma + \delta_s + \eta_t + \varepsilon_{ict}$$

where, $Year_t$ represent relative year dummies, defined relative to the year of Medicaid ACO implementation (t=0). The year immediately preceding implementation (t=-1) is omitted as the reference year.

The event window spans six years prior to the implementation (t=-6) through six years after implementation (t=6). This reflects the variation in implementation timing across states:

- For the last implementation cohort (2022), the earliest lead is t=-6 (corresponding to 2016)
- For the earliest implementation cohort (2017), the latest lag is t=6 (corresponding to 2023)

The lead term ($\sum_{t=-6}^{-2} \beta_t \cdot ACO_s \cdot Year_t$) captures and tests the common trends assumption by examining whether outcomes in states with and without Medicaid ACO diverged in the years before implementation. As shown in Figure 1, all estimated lead coefficients ($\beta_t, t < 0$) are not

© 2026 Jeung C et al. *JAMA Network Open*.

statistically different from zero, with 95% confidence intervals crossing the zero line on the x-axis. These results indicate that pre-implementation trends in outcomes were similar between states with and without Medicaid ACOs, supporting the validity of the common trends assumption.

© 2026 Jeung C et al. *JAMA Network Open*.

eFigure 1. Sample Restriction Criteria

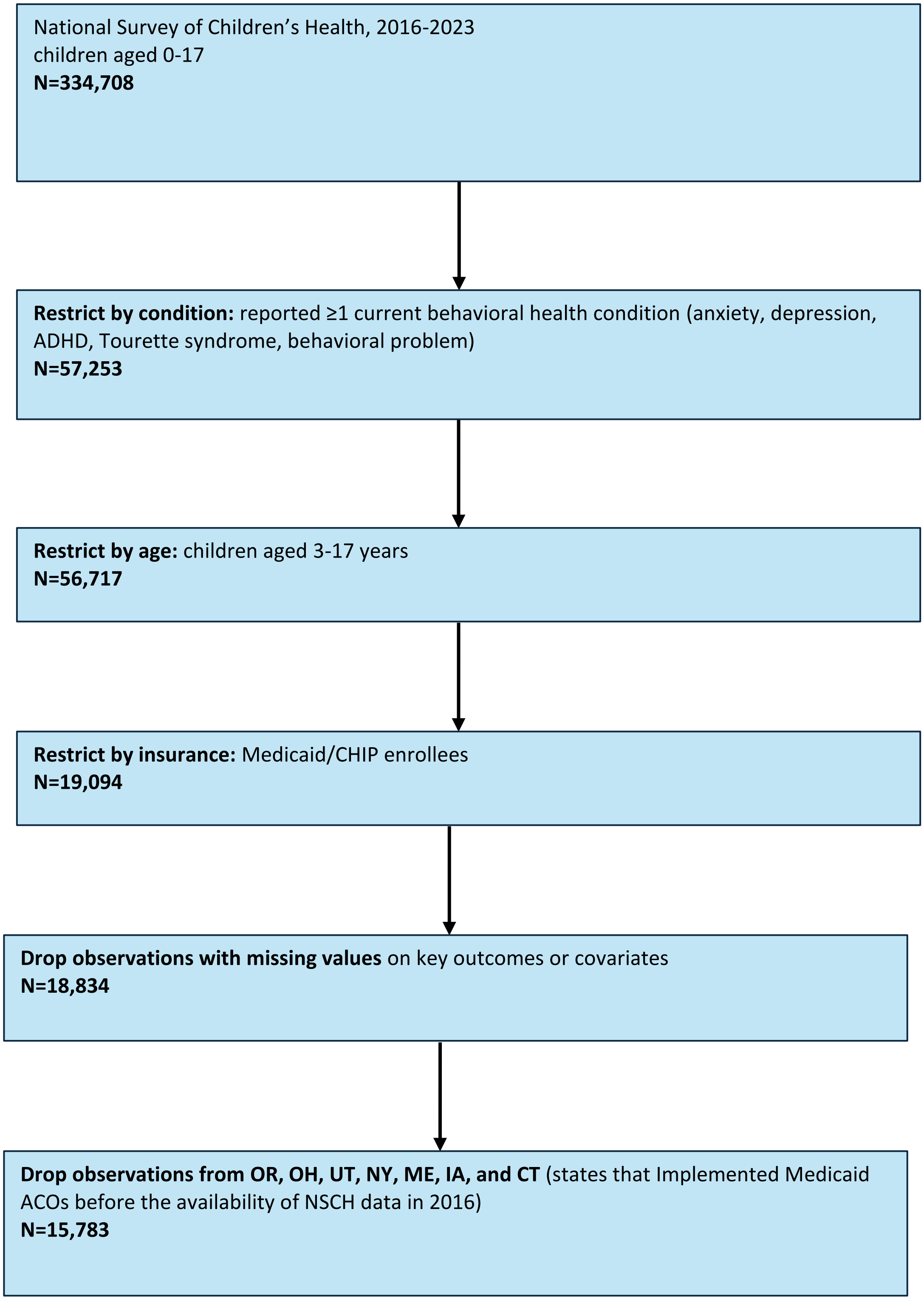

© 2026 Jeung C et al. *JAMA Network Open*.

eTable 1. Medicaid ACO Program Characteristics

State (Impl. Year)	Program Name	Contracting Mechanism	Payment Model	Downside Risk	BH in TCOC	BH Quality Metrics[a]	Enrollment Scope	Pediatric-Specific Features	HRSN / Social Risk Screening
Vermont (2017)	Vermont Medicaid Next Generation (VMNG); OneCare Vermont	Direct state–ACO contract; 1115 waiver (Global Commitment to Health); All-Payer Model Agreement (2017–2022; extended through 2024)†	Global capitation (all-inclusive population-based payment) + shared savings/losses; TCOC benchmark with risk corridor	Yes	Yes	Yes	Partial (~20–42% of Medicaid beneficiaries; voluntary provider participation)	None specified	Encouraged
Colorado[§] (2018)	Accountable Care Collaborative (ACC) Phase II; 7 Regional Accountable Entities (RAEs)	Direct state–RAE contract; mandatory enrollment; RAEs subcontract with PCMPs and BH providers	PMPM administrative payment + BH capitation; no shared savings/losses[a]	No	Yes	Yes[b]	Near-universal (~97% of Medicaid enrollees; mandatory assignment)	None specified	Required
Massachusetts (2018)	MassHealth ACO Program; Accountable Care Partnership Plans (ACO+MCO) and Primary Care ACO Plans	ACO contracts with MCO (Partnership Plans) or directly with state (Primary Care Plans); DSRIP 1115 waiver (2017–2022); extended 2022–2027	Shared savings + limited mandatory downside risk; primary care capitation (2022 extension); health equity incentive payments (2022+)[c]	Yes (mandatory but limited)	Yes[d]	Yes	~80%+ of eligible MassHealth members; statewide; mandatory enrollment	Yes — Increased focus on pediatric care coordination for children with complex needs (2022 waiver extension)	Required
Minnesota (2018)	Integrated Health Partnerships (IHP) 2.0; 26 IHPs serving 430,000+ enrollees (as of June 2020)	State contract with IHPs; MCOs required to work with IHPs; covers all MA and MinnesotaCare patients regardless of FFS/managed care status	Monthly population-based payment (clinical and social risk-adjusted); Track 1: upside-only shared savings; Track 2 (≥2,000 members): shared savings/losses	Track 2 only (negotiated per IHP)	Yes	Yes (negotiated per IHP)[e]	Statewide; all MA and MinnesotaCare patients (FFS and managed care)	None specified	Required
Rhode Island (2018)	Accountable Entities (AEs); 7 certified AEs; piloted 2016, full program July 2018	State certifies AEs; state requires MCOs to contract with AEs; 1115 waiver (Comprehensive Demonstration); MIIP infrastructure grants	TCOC-based; MCOs share up to 50% of savings with AEs based on quality performance; AEs may accept downside risk if OHIC-approved	No (initial phase; optional with OHIC approval; staged toward full risk)	Yes	Yes (common measure set; BH metrics govern share of savings)[g]	~70% of Medicaid beneficiaries; AEs contracted with state MCOs	Yes — 2Gen (two-generation) approach: SDOH screening for children under age 12 applied at household level; developmental screening for children under age 3 transitioned to P4P beginning PY4 (2021)	Required
New Jersey* (2020)	Regional Health Hub (RHH) Program; 4 designated RHHs (Camden Coalition; Trenton Health Team;	State-designated non-profit organizations; state appropriation funding; no MCO contract; RHHs	State appropriation only ($1.5–2.25M annually); no TCOC benchmark; no	N/A	N/A	N/A	Partial — 4 urban regions only; targeted Medicaid population	None specified	Encouraged

© 2026 Jeung C et al. *JAMA Network Open*.

State (Impl. Year)	Program Name	Contracting Mechanism	Payment Model	Downside Risk	BH in TCOC	BH Quality Metrics[a]	Enrollment Scope	Pediatric-Specific Features	HRSN / Social Risk Screening
	Greater Newark Healthcare Coalition; Health Coalition of Passaic County); established under P.L.2020, c.8 (A5977)	convene multi-sector partners and operate/use regional HIE; coordinate with state Office of Medicaid Innovation	shared savings or risk contract						
Delaware (2021)	Delaware Medicaid ACO Program; 4 authorized ACOs: Aledade Delaware ACO; Delaware Care Collaboration (Saint Francis Healthcare + Medical Society of Delaware); Delaware Children's Health Network (Nemours); Delaware Medicaid Quality Partners ACO (ChristianaCare)	ACO contracts directly with MCOs (AmeriHealth Caritas Delaware; Highmark Health Options); DMMA authorizes program; minimum 5,000 enrollees per contract	TCOC-based shared savings/losses	Yes	Yes	Yes (measure selection delegated to MCO-ACO contract; HEDIS/Delaware Common Scorecard alignment required)	Partial; voluntary ACO participation; minimum 5,000 enrollees per contract	Yes — Delaware Children's Health Network (Nemours) is a dedicated pediatric ACO (1 of 4 authorized ACOs)	Encouraged
Idaho** (2022)	Healthy Connections Value Care (HCVC); 11 VCOs at launch; two types: APCOs (≥1,000 enrollees) and AHCOs (≥10,000 enrollees); FQHC-centered	Direct state–VCO contract; FQHC-centered; state selects 7 quality measures annually with VCOs; FFS + PMPM care management fee	FFS + PMPM care management fee; TCOC-based shared savings/losses (physical health only)	Yes (APCOs: capped; AHCOs: uncapped)	No (physical health TCOC only; BH excluded)	No BH-specific measures	Partial — 11 VCOs at launch; FQHC-based; rural focus; statewide FQHC network (CHCN) as APCO	None specified	None

Notes

Abbreviations: ACO = Accountable Care Organization; AE = Accountable Entity (Rhode Island); AHCO = Accountable Hospital Care Organization (Idaho); APCO = Accountable Primary Care Organization (Idaho); BH = Behavioral Health; CHCN = Community Health Centers of Idaho (statewide FQHC network); CMHC = Community Mental Health Center; DMMA = Delaware Division of Medicaid and Medical Assistance; DSRIP = Delivery System Reform Incentive Payment; FFS = Fee-for-Service; FQHC = Federally Qualified Health Center; HCVC = Healthy Connections Value Care (Idaho); HEDIS = Healthcare Effectiveness Data and Information Set; HRSN = Health-Related Social Needs; IHP = Integrated Health Partnership (Minnesota); KPI = Key Performance Indicator; MA = Medical Assistance (Minnesota Medicaid); MCO = Managed Care Organization; MIIP = Medicaid Infrastructure and Investment Program (Rhode Island); OHIC = Office of the Health Insurance Commissioner (Rhode Island); PCMP = Primary Care Medical Provider (Colorado); PMPM = Per Member Per Month; RAE = Regional Accountable Entity (Colorado); RHH = Regional Health Hub (New Jersey); SDOH = Social Determinants of Health; SIM = State Innovation Model; TCOC = Total Cost of Care; VCO = Value Care Organization (Idaho); VMNG = Vermont Medicaid Next Generation; VBP = Value-Based Payment.

§ **Colorado:** Colorado ACC Phase 1 (2011) excluded; Phase 2 (July 2018) meets study ACO criteria based on TCOC accountability, integrated BH, and performance-based payment structure. https://www.coloradohealthinstitute.org/research/ways-raes

*** New Jersey:** New Jersey's implementation year reflects the Regional Health Hub (RHH) program, established under P.L.2020, c.8 (signed February 2020). RHHs are state-funded, multi-sector community health coordination entities and do not operate under a TCOC-based financial accountability mechanism; there is no shared savings arrangement, downside risk contract, or payment-linked quality metric structure. Downside Risk, BH in TCOC, and BH Quality Metrics are therefore coded N/A. New Jersey operated a prior Medicaid ACO Demonstration Project (P.L.2011, c.114) from 2012 to 2019, but this demonstration served a small, geographically concentrated population (4 urban regions, voluntary enrollment of high-utilization Medicaid beneficiaries) and was not considered for inclusion as a treatment state during the study period. New Jersey is excluded from sensitivity analyses.

© 2026 Jeung C et al. *JAMA Network Open*.

† Vermont All-Payer Model Agreement: The original All-Payer Model Agreement (APM) between Vermont and CMMI covered 2018–2022. In late 2022, Vermont and CMMI agreed to a one-year extension (2023), with an additional optional transition year (2024). The VMNG Medicaid ACO program and the associated 1115 waiver (Global Commitment to Health) have continued under both extensions. Vermont's 1115 waiver received five-year extensions approved by CMS in 2018 and 2022.

**** Idaho:** BH services are administered through a separate Pre-Paid Inpatient Health Plan (PIHP) and are excluded from the HCVC total cost of care scope. Idaho's HCVC program launched formally January 1, 2022 (State Plan Amendment ID-21-0002), following a pilot period beginning July 2020.

HRSN / Social Risk Screening — Classification Criteria:

The classification reflects whether HRSN or social risk screening was formally required as a condition of program participation, certification, or contract — regardless of whether screening performance was tied to payment.

Required: The state ACO program explicitly mandated HRSN or social risk screening or a related SDOH intervention through a contractual obligation, RFP evaluation criterion, or certification standard. Applicable to Massachusetts (annual HRSN screening required of all ACOs since 2018; screening performance factored into ACO payment — P4P-linked), Rhode Island (HRSN screening capacity required for AE certification; screening tied to P4P beginning PY4/2021 — P4P-linked), Colorado (SDOH strategy required as one of 29 RAE RFP evaluation criteria; CBO partnerships required by contract — not P4P-linked), and Minnesota (at least one SDOH/health equity intervention required in contract negotiations; social risk factors incorporated into payment risk adjustment — not P4P-linked).

Encouraged: The state ACO program incorporated SDOH or social risk strategies at a programmatic or aspirational level — through program design, authorization language, or model-level expectations — but did not mandate screening or a SDOH intervention as a condition of participation, certification, or contract. Applicable to Vermont (SDOH community investment budget required; ACO-level screening adopted by consensus, not mandate), New Jersey (SDOH coordination central to RHH model design; no contractual requirement), and Delaware (DHSS authorization cited social needs as ACO targets; no mandated screening tool or contractual requirement documented during study period).

None specified: The state ACO program did not document any statewide mandate, contractual requirement, or programmatic expectation specifically directed at HRSN or social risk screening. Applicable to Idaho, where the HCVC program excluded BH from its TCOC scope and referenced SDOH community investments only aspirationally.

[a] **BH Quality Metrics** listed here reflect standard performance measures and do not include quality metrics specifically designed for or restricted to pediatric population.

[b] **Colorado:** RAEs receive a $15.50 PMPM administrative payment (of which $4.00 is withheld pending KPI performance) plus a separate BH capitation rate and BH incentive payments (up to 5% of BH capitation). RAEs do not participate in traditional shared savings/losses on TCOC but receive incentives through KPI performance instead.

[c] **Massachusetts Payment:** The first waiver period (2017–2022) did not include mandatory downside risk in the initial ACO contract structure; mandatory but limited downside risk was a feature of the extended waiver (2022–2027). The 2022 extension also added primary care capitation and health equity incentive payments.

[d] **Massachusetts BH in TCOC:** Under Partnership Plans, BH is included in the full network. Under Primary Care ACO Plans, BH is contracted via the Massachusetts Behavioral Health Partnership. BH integration requirements differ between plan types.

[e] **Minnesota BH Quality Metrics:** BH quality measures are negotiated individually per IHP (e.g., Alcohol and Other Drug (AOD) initiation/engagement, MH follow-up post-discharge, depression screening/follow-up, antidepressant/antipsychotic adherence etc.).

[g] **Rhode Island BH Quality Metrics:** BH measures are drawn from the State Innovation Model common measure set and govern the share of savings distributed to AEs. Measures include follow-up after mental health inpatient discharge (7- and 30-day), AOD initiation and engagement, antidepressant medication management, and depression screening and follow-up.

References

Vermont.

- Department of Vermont Health Access (DVHA). Vermont Medicaid Next Generation (VMNG) Accountable Care Organization Program. https://dvha.vermont.gov/initiatives/payment-reform/vermont-medicaid-next-generation-aco-program
- Department of Vermont Health Access (DVHA). OneCare Vermont ACO Contract and Exhibits (signed March 23, 2018). https://dvha.vermont.gov/sites/dvha/files/documents/Administration/1onecare-aco-32318-3-final-signed-with-exhbits.pdf
- Centers for Medicare & Medicaid Services (CMS). Vermont All-Payer ACO Model. https://www.cms.gov/priorities/innovation/innovation-models/vermont-all-payer-aco-model
- Green Mountain Care Board. All-Payer Model: Frequently Asked Questions [includes APM extension 2023–2024]. https://gmcboard.vermont.gov/all-payer-model/frequently-asked-questions
- Delbanco S, Hoo E. Vermont's Bold Experiment in Community-Driven Health Care Reform. Commonwealth Fund. May 2018. https://www.commonwealthfund.org/publications/case-study/2018/may/vermonts-bold-experiment-community-driven-health-care-reform

© 2026 Jeung C et al. *JAMA Network Open*.

- Center for Health Care Strategies (CHCS). Addressing Social Determinants of Health through Medicaid Accountable Care Organizations. May 2019. https://www.chcs.org/addressing-social-determinants-health-medicaid-accountable-care-organizations/

Colorado.

- Colorado Department of Health Care Policy & Financing (HCPF). Accountable Care Collaborative Phase II: Provider and Stakeholder Resource Center. https://hcpf.colorado.gov/accountable-care-collaborative-phase-ii-provider-and-stakeholder-resource-center
- Colorado HCPF. Key Performance Indicator Methodology FY22–23. https://hcpf.colorado.gov/sites/hcpf/files/Key%20Performance%20Indicator%20Methodology%20FY22-23.pdf
- National Academy for State Health Policy (NASHP). Three States' Strategies to Improve Behavioral Health Services Delivery through Medicaid Accountable Care Programs [includes Colorado ACC Phase II detail]. 2020. https://nashp.org/three-states-strategies-to-improve-behavioral-health-services-delivery-through-medicaid-accountable-care-programs/
- Center for Health Care Strategies (CHCS). Addressing Social Determinants of Health through Medicaid Accountable Care Organizations [SDOH in RAE RFP evaluation criteria]. May 2019. https://www.chcs.org/addressing-social-determinants-health-medicaid-accountable-care-organizations/

Massachusetts.

- Browne T, Tonn B, Bhatt A, et al. Association of a Medicaid Accountable Care Organization Program With Cost, Utilization, and Quality of Care. *JAMA Network Open*. 2023;6(5):e2314791.
- Centers for Medicare & Medicaid Services. MassHealth Section 1115 Demonstration: Flexible Services Protocol Approval Letter. October 4, 2018. https://www.medicaid.gov/Medicaid-CHIP-Program-Information/By-Topics/Waivers/1115/downloads/ma/MassHealth/ma-masshealth-flex-srvcs-prtcl-appvl-20181004.pdf
- Blue Cross Blue Shield of Massachusetts Foundation. What to Know Now About MassHealth ACOs. October 2023. https://www.bluecrossmafoundation.org/sites/g/files/csphws2101/files/2023-10/ACO%20Primer_2023_FINAL.pdf
- MassHealth. Information for MassHealth ACOs and HRSN Providers [includes Flexible Services Program and HRSN Supplemental Services]. https://www.mass.gov/info-details/information-for-masshealth-acos-and-hrsn-providers

Minnesota.

- State Health Access Data Assistance Center (SHADAC). Integrated Health Partnerships — Minnesota's Medicaid Accountable Care Organization Model. University of Minnesota. 2024. https://www.shadac.org/news/integrated-health-partnerships-minnesota-medicaid-accountable-care-organization
- Minnesota Department of Human Services (DHS). Integrated Health Partnerships RFPs and Contracts [2025 RFP includes Track 1/2 structure and SDOH requirements]. https://mn.gov/dhs/partners-and-providers/grants-rfps/integrated-health-partnerships/
- Center for Health Care Strategies (CHCS). Medicaid ACOs Version 2.0 Underway in Minnesota and Colorado [IHP 2.0 launch January 2018]. 2018. https://www.chcs.org/medicaid-accountable-care-organizations-version-2-0-underway-minnesota-colorado/
- Center for Health Care Strategies (CHCS). Prioritizing Social Determinants of Health in Medicaid ACO Programs: A Conversation with Two Pioneering States [interview with Mat Spaan, MN DHS]. 2019. https://www.chcs.org/prioritizing-social-determinants-health-medicaid-aco-programs-conversation-two-pioneering-states/

Rhode Island.

- Rhode Island EOHHS. Accountable Entity Certification Standards, Program Year 4 [SDOH screening P4P, developmental screening P4P, 2Gen household-level approach]. March 2021. https://eohhs.ri.gov/sites/g/files/xkgbur226/files/2021-03/Attachment_H_PY4_AE_Certification_Standards_.pdf
- Rhode Island EOHHS. TCOC Quality Measures and P4P Methodology Implementation Manual [QPY7–8; full BH quality measure set and P4P scoring methodology]. https://eohhs.ri.gov/media/47696/download?language=en
- Crumley D. Rhode Island's Accountable Entities Emphasize Children's Health and Social Needs [2Gen framework; developmental screening for children under age 3 transitioned to P4P in PY4/2021]. NASHP. 2021. https://nashp.org/rhode-islands-accountable-entities-emphasize-childrens-health-and-social-needs/
- Higgins E. Q&A: How Rhode Island Tackles Social Determinants of Health through Its Accountable Entity Model [MIIP grants, HRSN certification requirements, AE program structure]. NASHP. November 2018. https://nashp.org/qa-how-rhode-island-tackles-social-determinants-of-health-through-its-accountable-entity-model/

New Jersey.

- New Jersey P.L.2020, c.8 (A5977). An Act establishing a Regional Health Hub Program as a replacement to the Accountable Care Organization Demonstration Project. Signed February 2020. https://pub.njleg.gov/bills/2018/A9999/5977_R2.PDF
- Camden Coalition of Healthcare Providers. Regional Health Hub Program Overview. https://camdenhealth.org/work/regional-health-hub/

© 2026 Jeung C et al. *JAMA Network Open*.

- New Jersey Health Care Quality Institute. A Roadmap to Redesign the New Jersey Medicaid ACOS to Form Regional Health Hubs https://www.njhcqi.org/wp-content/uploads/2019/06/NJHCQI_RHHRoadMap_Report_FINAL.pdf
- Healthcare Innovation. N.J. Legislation Creates Four Regional Health Hubs. 2020. https://www.hcinnovationgroup.com/interoperability-hie/health-information-exchange-hie/news/21122244/nj-legislation-creates-four-regional-health-hubs
- New Jersey P.L.2011, c.114. Medicaid Accountable Care Organization Demonstration Project Act [prior program; not included as treatment state due to small, geographically concentrated population]. https://pub.njleg.gov/bills/2010/PL11/114_.pdf

Delaware.

- State of Delaware, DHSS/DMMA. DHSS Authorizes Four Medicaid Accountable Care Organizations [program summary; 4 ACO names; contracts begin July 1, 2021 through December 2024]. News release. September 22, 2020. https://news.delaware.gov/2020/09/22/dhss-authorizes-four-medicaid-accountable-care-organizations/
- Centers for Medicare & Medicaid Services. Diamond State Health Plan Section 1115 Demonstration: 2020 Annual and 4th Quarterly Report [MCO/ACO contracts begin July 1, 2021; TCOC payment structure; Track 1/2 risk arrangement]. https://www.medicaid.gov/medicaid/section-1115-demonstrations/downloads/de-dshp-annl-rpt-jan-dec-2020.pdf
- Centers for Medicare & Medicaid Services. Diamond State Health Plan Section 1115 Demonstration: 2022 3rd Quarterly Report [ongoing ACO program status; TCOC scope; second ACO cohort CY2022]. https://www.medicaid.gov/medicaid/section-1115-demonstrations/downloads/de-dshp-qrtly-monitor-rpt-jul-sep-2022.pdf

Idaho.

- Centers for Medicare & Medicaid Services. Idaho State Plan Amendment ID-21-0002 [Healthy Connections Value Care Program; APCO/AHCO structure; FFS + PMPM + shared savings/losses; effective January 1, 2022]. https://www.medicaid.gov/medicaid/spa/downloads/ID-21-0002.pdf
- National Academy for State Health Policy (NASHP). Idaho Develops a Medicaid Value-Based Payment Model for Its FQHCs, Based on Cost and Quality [HCVC program design; VCO types; FQHC role; quality measure structure]. https://nashp.org/idaho-develops-a-medicaid-value-based-payment-model-for-its-fqhcs-based-on-cost-and-quality/
- Idaho DHW. DHW's Living Strategic Plan: A Year of Progress [confirms HCVC went live January 1, 2022; performance data collection 2022; Medicaid Expansion population added in PY2]. https://healthandwelfare.idaho.gov/dhw-voice/dhws-living-strategic-plan-year-progress-toward-ensuring-affordable-available-healthcare

© 2026 Jeung C et al. *JAMA Network Open*.

eTable 2. Baseline (Pre-Medicaid ACO Implementation) Outcome Rates in States with and without Medicaid ACO

	States without Medicaid ACO (%)	States with Medicaid ACO (%)	Differences in Percentage Points	P-value
Had a personal doctor/nurse	72.87	78.35	5.47	0.072
Received treatment from a mental health professional	46	52.9	6.9	0.062
Unmet mental health needs	5.55	3.81	-1.74	0.2
Received coordinated care	54.85	51.46	-3.39	0.413
Received family centered care	63.68	72.09	8.41	0.01

Notes: Values represent weighted percentages. For Medicaid ACO states, the pre-period includes survey years prior to each state's first ACO implementation. For states without Medicaid ACO, the pre-period includes never-exposed states observed in the same calendar-year range as the exposed states' pre-period. P-values are from survey-weighted tests of differences in means. Estimates are weighted to be nationally representative.

© 2026 Jeung C et al. *JAMA Network Open*.

eTable 3. Association between Medicaid ACO Implementation and Behavioral Health Diagnosis and Condition Severity

	DID Estimate (95% CI)	P-value
Panel A: Any Behavioral Health Condition (Full Medicaid & CHIP Sample)		
Any Behavioral Health Condition	-0.35 (-6.18, 5.49)	0.91
Panel B: Condition Severity (Children with a Behavioral Health Condition)		
Any Severe Behavioral Health Condition	-0.26 (-6.72, 7.23)	0.94

Notes: Estimates are from staggered difference-in-differences models using National Survey of Children's Health data. Panel A includes all Medicaid and CHIP children aged 3-17 and tests whether Medicaid ACO implementation predicts having any reported behavioral health condition. Panel B is restricted to children with behavioral health condition and tests whether Medicaid ACO implementation predicts reported condition severity. All models adjust for child age (categorical), sex, race and ethnicity, and year and state fixed effects. Models are survey-weighted to ensure national representativeness. Estimates are expressed in percentage points.

© 2026 Jeung C et al. *JAMA Network Open*.

eTable 4. Sensitivity Analyses

	(1) Fully Adjusted DID	(2) Unadjusted DID (State FE and Year FE)	(3) Without Severity	(4) Fully Adjusted + State-specific Linear Trends	(5) Excluding First Implementation Year	(6) Excluding Observations 2020-2021
Had a personal doctor/ nurse	-2.48 (-10.10, 5.14)	-2.23 (-9.93, 5.46)	-2.68 (-10.30, 4.94)	2.03 (-9.91, 13.96)	-3.88 (-11.99, 4.23)	-5.76 (-14.07, 2.55)
Received treatment from a mental health professional	-7.27 (-15.53, 0.98)	-6.26 (-15.46, 2.95)	-7.01 (-15.18, 1.17)	-0.74 (-13.40, 11.93)	-9.04* (-17.75, -0.34)	-9.30* (-18.08, -0.51)
Unmet mental health needs	4.76* (0.83, 8.70)	4.77* (0.78, 8.76)	4.66* (0.74, 8.59)	8.50* (0.66, 16.35)	3.90 (-0.19, 8.00)	5.13* (0.39, 9.88)
Received coordinated care	-5.17 (-15.57, 5.22)	-6.56 (-17.14, 4.02)	-5.1 (-15.41, 5.21)	-11.42 (-27.20, 4.37)	-4.09 (-15.19, 7.02)	-7.44 (-18.65, 3.77)
Received family-centered care	-0.46 (-8.29, 7.37)	-0.30 (-8.12, 7.51)	-0.59 (-8.45, 7.27)	4.37 (-6.96, 15.70)	-1.42 (-9.79, 6.94)	-1.04 (-9.56, 7.49)

Notes: Column (1) presents the fully adjusted difference-in-differences model. Column (2) includes only state and year fixed effects. Column (3) excludes condition severity from the fully adjusted difference-in-differences model. Column (4) additionally allows for state-specific linear time trends. Column (5) excludes the first year of Medicaid ACO implementation. Column (6) excludes observations for the peak of COVID-19 (2020-2021).

© 2026 Jeung C et al. *JAMA Network Open*.

eTable 5. Pre-Implementation Baseline Outcome Rates and Relative Percentage Changes Associated with Medicaid ACO

	Pre-period Baseline (%)	DID Estimate (Percentage points)	Relative Change (%)
Had a personal doctor/nurse	78.35	-2.48	-3.17
Received treatment from a mental health professional	52.9	-7.27	-13.74
Unmet mental health needs	3.81	4.76	124.93
Received coordinated care	51.46	-5.17	-10.05
Received family centered care	72.09	-0.46	-0.64

Notes: Pre-implementation baseline rates are calculated as survey-weighted means among children in exposed states during the pre-implementation period. Relative changes are calculated by expressing the difference-in-differences estimates (percentage points) as a percentage of the pre-policy baseline rate.

© 2026 Jeung C et al. *JAMA Network Open*.

eTable 6. Sensitivity Analysis: Difference-in-Differences Estimates using State-Clustered Standard Errors and Wild Cluster Bootstrap

	DID Estimate (95% CI)	Wild Cluster Bootstrap p-value
Had a personal doctor/nurse	−2.48 (−10.51, 5.55)	0.582
Received treatment from a mental health professional	−7.27 (−15.62, 1.07)	0.189
Unmet mental health needs	4.76 (1.03, 8.50)	0.016
Received coordinated care	−5.17 (−12.54, 2.19)	0.262
Received family-centered care	−0.46 (−10.61, 3.11)	0.382

Notes: Estimates are from fully adjusted two-way fixed effects difference-in-differences models. Models incorporate NSCH sampling weights and include state and year fixed effects. Because the exposure varies at the state level, robust standard errors are clustered by state. 95% confidence intervals are based on these state-clustered standard errors. To account for the small number of treated states, p-values are computed using a wild cluster bootstrap procedure (9,999 replications; Rademacher weights; clustering by state). Coefficients are reported in percentage points.

© 2026 Jeung C et al. *JAMA Network Open*.

eTable 7. Sensitivity Analyses: Excluding Structurally Distinct Medicaid ACO Models (Idaho and/or New Jersey)

	(1)<br>Main Model	(2)<br>Excluding Idaho	(3)<br>Excluding New Jersey	(4)<br>Excluding Idaho & New Jersey
Had a personal doctor/nurse	-2.48 (-10.10, 5.14)	-3.81 (-12.02, 4.40)	-4.13 (-11.45, 3.20)	-6.08 (-14.10, 1.95)
Received treatment from a mental health professional	-7.27 (-15.53, 0.98)	-7.09 (-16.04, 1.86)	-6.03 (-14.92, 2.86)	-5.58 (-15.48, 4.32)
Unmet mental health needs	4.76* (0.83, 8.70)	4.65* (0.41, 8.90)	5.65** (2.04, 9.25)	5.62** (1.71, 9.53)
Received coordinated care	-5.17 (-15.57, 5.22)	-4.90 (-16.11, 6.31)	-2.28 (-13.38, 8.81)	-1.51 (-13.75, 10.73)
Received family-centered care	-0.46 (-8.29, 7.37)	-4.14 (-13.14, 4.86)	-1.03 (-9.74, 7.68)	-1.15 (-10.72, 8.43)

Notes: **$p<0.01$, * $p<0.05$. Data from the 2016–2023 National Survey of Children's Health. Linear probability models were estimated, controlling for child age (categorical), sex, race and ethnicity, autism, specific behavioral health condition (Tourette syndrome, anxiety, depression, behavioral problem, ADHD) and severity for each (severe vs. not severe), as well as year and state fixed effects. Reported estimates are expressed in percentage points, and all models are weighted to ensure national representativeness.

© 2026 Jeung C et al. *JAMA Network Open*.
