# Supplementary material for: Medicaid Accountable Care Organization Implementation and Behavioral Health Care for Children
Source: JAMA Netw Open. 2026 Apr 23;9(4):e268890. doi: 10.1001/jamanetworkopen.2026.8890 (PMC13107223; doi:10.1001/jamanetworkopen.2026.8890)
Supplement: Supplement 2. — Data Sharing Statement [file jamanetwopen-e268890-s002.pdf]

Data Sharing Statement

Jeung. Medicaid Accountable Care Organization Implementation and Behavioral Health Care for Children. *JAMA Netw Open*. Published April 23, 2026.
doi:10.1001/jamanetworkopen.2026.8890

Data
Data available: Yes
Data types: Deidentified participant data
How to access data: The study used publicly available, de-identified data from the National Survey of Children's Health (NSCH), administered by the U.S. Census Bureau and the Health Resources and Services Administration's Maternal and Child Health Bureau. Data and documentation, including the data dictionary, are available at the Data Resource Center for Child and Adolescent Health (https://www.childhealthdata.org/)
When available: With publication

Supporting Documents
Document types: Statistical/analytic code
How to access documents: The Stata code used in this analysis is available from the corresponding author upon request (cjeung@albany.edu)
When available: With publication

Additional Information
Who can access the data: Anyone requesting the data.
Types of analyses: For any research purpose.
Mechanisms of data availability: Without investigator support; data are publicly available through the original data source upon request or direct download.
